# Combination of Low-Temperature Electrosurgical Unit and Extractive Electrospray Ionization Mass Spectrometry for Molecular Profiling and Classification of Tissues

**DOI:** 10.3390/molecules24162957

**Published:** 2019-08-15

**Authors:** Gennady Sukhikh, Vitaliy Chagovets, Xinchen Wang, Valeriy Rodionov, Vlada Kometova, Alisa Tokareva, Alexey Kononikhin, Natalia Starodubtseva, Konstantin Chingin, Huanwen Chen, Vladimir Frankevich

**Affiliations:** 1 National Medical Research Center for Obstetrics, Gynecology and Perinatology named after Academician V.I.Kulakov of the Ministry of Healthcare of Russian Federation, Moscow 117997, Russia; 2 Department of Obstetrics, Gynecology, Perinatology and Reproductology, First Moscow State Medical University named after I.M. Sechenov, Moscow 119991, Russia; 3 Jiangxi Key Laboratory for Mass Spectrometry and Instrumentation, East China University of Technology, Nanchang 330013, China; 4 Moscow Institute of Physics and Technology, Moscow 141701, Russia

**Keywords:** molecular imaging, mass spectrometry, EESI, breast cancer, intraoperative navigation

## Abstract

Real-time molecular navigation of tissue surgeries is an important goal at present. Combination of electrosurgical units and mass spectrometry (MS) to perform accurate molecular visualization of biological tissues has been pursued by many research groups. Determination of molecular tissue composition at a particular location by surgical smoke analysis is now of increasing interest for clinical use. However, molecular analysis of surgical smoke is commonly lacking molecular specificity and is associated with significant carbonization and chemical contamination, which are mainly related to the high temperature of smoke at which many molecules become unstable. Unlike traditional electrosurgical tools, low-temperature electrosurgical units allow tissue dissection without substantial heating. Here, we show that low-temperature electrosurgical units can be used for desorption of molecules from biological tissues without thermal degradation. The use of extractive electrospray ionization technique for the ionization of desorbed molecules allowed us to obtain mass spectra of healthy and pathological tissues with high degree of differentiation. Overall, the data indicate that the described approach has potential for intraoperative use.

## 1. Introduction

In computer-assisted surgery, the surgeon’s manipulations are largely guided by a navigation system. There is great interest in using mass spectrometry (MS)-based navigation because MS allows molecular visualization of operated tissues.

Mass spectrometry molecular imaging by matrix-assisted laser desorption ionization (MALDI) is a useful method in tissue-based studies [1,2], but tissue sample preparation is required. Laser desorption methods [3], including picosecond infrared laser desorption method [4,5], do not need a preanalytical stage, which makes analysis less time-consuming. One of the most popular MS methods for tissue analysis is desorption electrospray ionization mass spectrometry (DESI-MS), in which tissue molecules are desorbed from their surface by electrosprayed solvent droplets (water, methanol, acetonitrile, etc.). [6] This method is good in lipid marker detection of tumor tissue [7], demonstrating more than 90% accuracy of margin in tissue detection [8]. Tissue-spray method [9], in which voltage is applied directly on tissue sample, has similar diagnostic efficiency with DESI [10] and can also be applied for offline tissue analysis [11,12]. However, the above methods are not directly suitable for intraoperative analysis.

The MassSpec Pen technique, based on liquid surface extraction method [13,14], has been demonstrated for tissue diagnosis and differentiation with minimal tissue damage [15]. Rapid evaporative ionization mass spectrometry (REIMS) is another method of molecular visualization during electrosurgery [16], with diagnostic accuracy of about 95% [17,18]. Electrosurgery is now used in almost every surgical procedure [19,20]. It is used both to cut tissue and to control bleeding by coagulating the blood vessels. Typically, electrosurgery produces surgical smoke. The chemical composition of smoke produced by high-frequency electrosurgery dates back to 1995 [21]. During the tissue incision with high-frequency current, the molecules at the incision area evaporate and, together with water and possible soot particles, form surgical smoke. Electrosurgical smoke mainly contains hydrocarbons, nitriles, fatty acids, and phenols [22,23,24]. However, the smoke not only contains simple gaseous mixtures but also cell metabolites and therefore possible biomarkers for different diseases like cancer or bacterial infection. Along with neutral molecules, surgery smoke has also been demonstrated to contain gas-phase ions, which can be analyzed directly without auxiliary ionization, such as with REIMS [25,26]. Thus, the analysis of surgical smoke can be used in intraoperative medical diagnostics. Molecular analysis of smoke may not always provide complete information about the origin of a tissue under investigation, which is related to the high temperature of smoke at which many molecules become unstable. Also, the analysis of surgical smoke is commonly complicated by the formation of soot particles and other contamination. It is possible to increase the efficiency of smoke analysis by MS by lowering its temperature.

The use of low-temperature electrosurgery units (LTEU) avoids the formation of soot particles. In the case of LTEU, the increase in tissue temperature does not exceed 45 °C for the cut-off mode (unmodulated radiation) and 65 °C for the coagulation regimen [27,28]. This is dramatically different with low-frequency methods and essentially reduces the carbonization of the tissues to zero. Low-temperature desorption allows extraction of intact molecules from biological tissues. At the same time, lowering the temperature may result in decreasing the efficiency of ion formation [29], and assistance for ionizing neutral molecules is required. In the present study, the aerosol released by soft LTEU desorption was directly analyzed using extractive electrospray ionization mass spectrometry (EESI-MS). EESI is an electrospray-based ionization technique that permits soft ionization of biological molecules in complex chemical matrices, including aerosols such as surgical smoke or human breath [30]. A similar method has previously been shown to increase mass spectrometric signal, especially the signal of structural phospholipids [26]. Moreover, EESI can potentially be used for selective extraction and analysis of compounds that belong to a certain class [31].

The results indicate the high potential of this approach for the sensitive molecular analysis of biological tissues.

## 2. Materials and Methods

**Figure 1:**
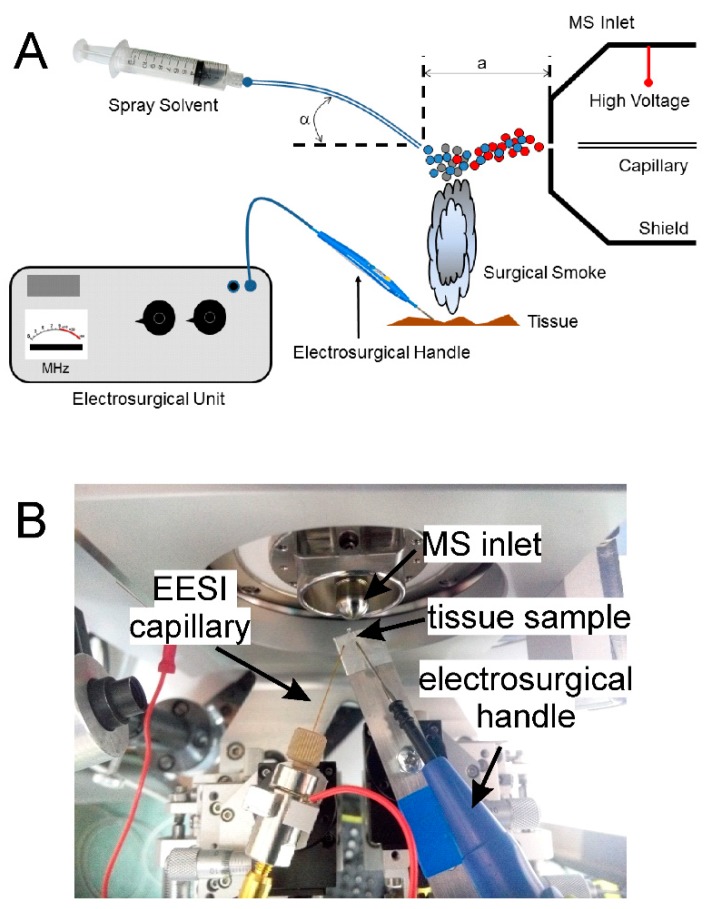
The schematic setup (**A**) and a photo of the ionization source (**B**).

Methanol and water, both of HPLC grade, were purchased from Sigma-Aldrich (St. Louis, MO, USA). The LTEU-EESI-MS setup was based on the commercially available quantum molecular resonance (QMR) electrosurgery unit (Vesalius, Telea Inc., Vicenza, Italy) and the Maxis Impact (Bruker Daltonics, Bremen, Germany) mass spectrometer with a home-built EESI source (Figure 1). A low-frequency electrosurgical unit, Bovie A1250S-V (475 kHz, 80 W), (Apyx Medical, Antioch, TN, USA) was used for comparative analysis. Testo 875 thermal (testo, Lenzkirch, Germany) imager was used for the tissue’s temperature measurement. The power supplied by the QMR generator to the scalpel was 40 W. The voltage on the electrospray source was set to 0 V, while the cap electrode of the mass spectrometer was set to −4000 V. The distance between the edge of the sprayer and the mass spectrometer inlet was about 0.5 cm (“a” in Figure 1A). The measurements were carried out in the range of *m/z* 100–1500 in positive-ion mode. The EESI solvent was methanol/water (90/10, *v*/*v*).

Chicken meat from a grocery store was used as sample for the test and method optimization. The breast cancer experiments were carried out on biopsy materials from patients treated at the National Medical Research Center for Obstetrics, Gynecology and Perinatology named after academician V.I. Kulakov of the Ministry of Healthcare of the Russian Federation (Moscow, Russia). All clinical investigations were conducted according to the principles expressed in the Declaration of Helsinki. All patients read and signed informed consents approved by the ethical committee of the National Medical Research Center for Obstetrics, Gynecology and Perinatology named after academician V.I. Kulakov. The samples were sliced for the histological study, and the rest were frozen in liquid nitrogen and stored under −75 °C until the investigation. Microscopic samples were investigated by optical microscopy using an Olympus MX51 light microscope (Olympus Corporation, Tokyo, Japan). A slice of a sample adjacent to the histologically investigated region was cut, thawed, and fixed on the holder in the ion source for the analysis. Biopsy samples of healthy tissues and malignant tumors separated by a histologist were taken from 50 patients for the development of a classification model. Five samples of tissues with tumor and bordered normal tissues characterized by the histologist were taken for testing the classification model and to calculate sensitivity and specificity of the model. Mass spectra were recorded from about 20 different points of each of the five samples. Thus, a set of 100 mass spectra obtained from 5 tissue samples with both normal and cancer regions were used to validate the classification model.

The resulting mass spectrometric data were transformed into a table of peak intensities using a set of functions developed in the R language, with columns corresponding to *m/z* values of the detected peaks and rows corresponding to individual samples. Subsequently, the tabulated data was aligned by the average value, scaled by the Pareto method, and analyzed using the orthogonal partial least squares discriminant analysis (OPLS-DA) method. The data on the contribution of each peak obtained from the OPLS-DA model was then used to calculate the “estimated parameter” of tissue pathology at a separately analyzed point.

The lipid nomenclature used throughout the paper is in accordance with LIPID MAPS [32] terminology and shorthand notation summarized in [33].

## 3. Results and Discussion

**Figure 2:**
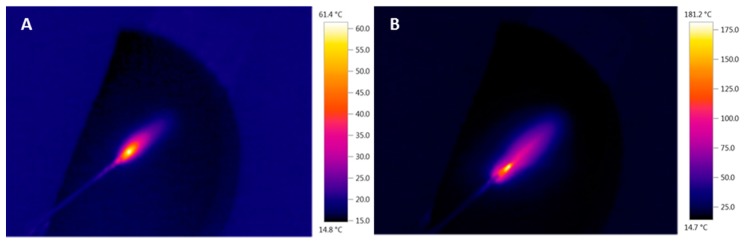
Cutting of chicken tissue with low-temperature electrosurgery unit (**A**) and low-frequency electrosurgery unit (**B**).

Chicken meat samples were used to study the properties and possibilities of the method under development and for the performance tuning of the experimental setup. Vesalius QMR surgical generator was used to provide desorption in the current study. This surgical instrument is considered as a low-temperature one. Indeed, the temperature in the electrosurgery unit probe region, measured by thermal imager, was about 61.4 °C on the chicken skin (Figure 2A). The temperature of the same sample was about 180 °C when the low-frequency electrosurgery unit was used (Figure 2B). The low temperature in the heating zone is a big advantage, both for the patient and for mass spectrometric analysis. Significant advantages include reducing tissue damage due to less heating, avoiding the formation of soot particles, preserving the native form of most biomolecules, and more precise positioning due to a reduction in the area from which ions are generated. However, because of low temperature, the efficiency of ion formation in the process of cutting or coagulation decreases [29]. Post-ionization techniques, which has already been shown as an efficient approach [26], can be implemented to enhance the ion yield in such cases.

For the soft ionization of aerosol desorbed during LTEU desorption, we used EESI. In EESI, the neutral aerosol is crossed by electrospray plume to produce analyte ions in front of a mass spectrometer (Figure 1A). This design allows the analysis of complex biological samples with minimal pretreatment [30,34].

**Figure 3:**
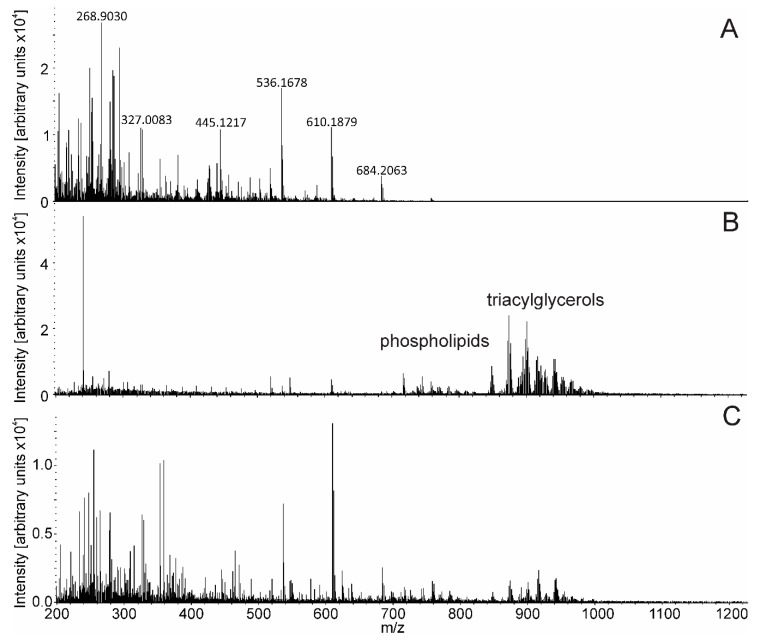
Low-temperature electrosurgery unit mass spectrometry (LTEU-MS) spectrum (**A**), LTEU- extractive electrospray ionization (EESI)-MS spectrum (**B**) and a mass spectrum obtained with high-temperature electrosurgery unit (**C**). Chicken skin was used as sample.

The spectrum in Figure 3A shows the absence of an informative signal when an EESI solvent was not supplied, demonstrating that ions were not generated from a tissue in the LTEU desorption process. The peaks in this mass spectrum can be identified as common mass spectrometry contaminants, such as ammonium adducts of polysiloxanes (within 10 ppm accuracy) [35]. A good-quality spectrum of tissue was only achieved when the EESI solution was supplied (Figure 3B). The most intensive peaks were grouped in two clusters. The first cluster was in the *m/z* range of 600–800 and identified as phospholipids according to accurate mass within 10 ppm (lysophosphatidylethanolamines (LPE), phosphatidylethanolamines (PE), phosphatidylcholines (PC)). The second cluster was in the *m/z* range of 800–1000 and identified as triacylglycerols according to accurate mass within 10 ppm (Table S1). The difference between the LTEU-EESI-MS spectrum and the mass spectrum obtained with high-temperature electrosurgery unit is evident from Figure 3B,C. Peaks in the higher mass range are less intensive in Figure 3C and more intensive in lower masses, which can be a consequence of thermal degradation.

Signal from a set of lipids, including PC 34:2, PC 34:1, PC 34:0, TG 52:3, TG 54:4, and TG 57:5, was used for the LTEU-EESI-MS ion source optimization. Several important parameters, including the EESI solvent composition (the optimal ratio is methanol/water 9/1, *v*/*v*), power of the surgical probe (35–40W), and the proper geometry of the setup were determined for subsequent analyses during the experiments with chicken meat samples (Figure S1).

Breast cancer samples were used for method validation after its optimization. The possibility to distinguish between normal and cancer tissues was tested.

**Figure 4:**
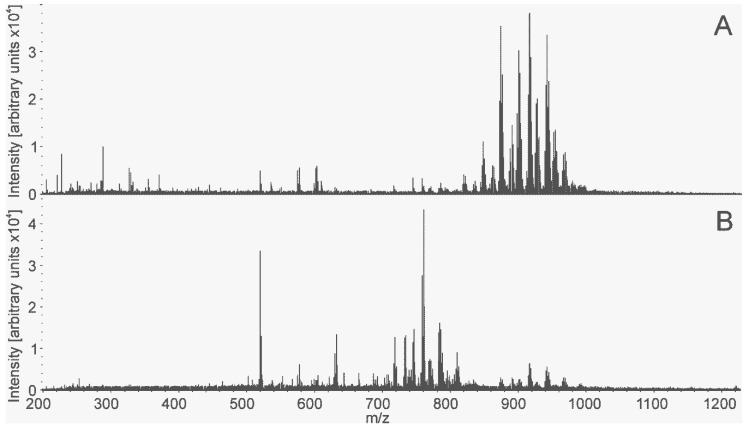
Positive-ion mass spectra of normal tissue (**A**) and cancer tissue (**B**) generated by LTEU-EESI-MS.

The mass spectra in Figure 4 demonstrate differences not only in the intensity of peaks but also in the presence of some peaks unique for the pathology.

**Figure 5:**
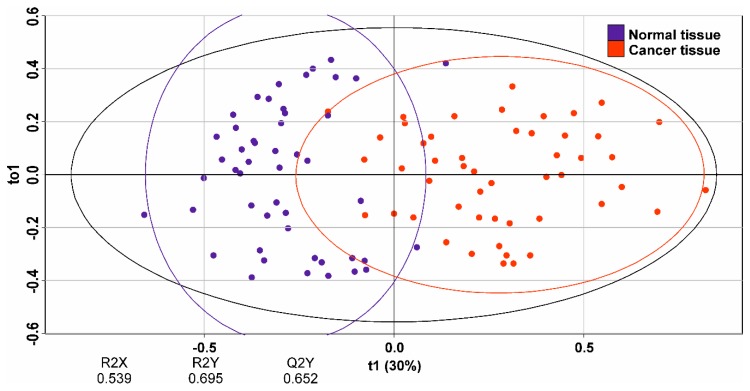
Scores plot of the orthogonal projections to latent structures discriminant analysis (OPLS-DA) model for separation of pathological (red dots) and normal (blue dots) tissues.

The most profound difference between the mass spectra was observed in the *m/z* range of 600–1000. These peaks were mostly identified as glycero- and phospholipids according to accurate mass within 10 ppm. Supervised orthogonal projections to latent structures discriminant analysis (OPLS-DA) was applied to the mass spectrometric data on histologically validated samples to create a classification model. The value of −1 was set for normal tissue and 1 for cancer. The model confirmed significant distinction between normal and cancer LTEU-EESI-MS profiles. Data points corresponding to these two groups of tissues were clustered separately on the OPLS-DA score plot (Figure 5). The developed statistical model showed good potential for classification between normal and cancer tissues scored with Q^2^ = 0.65. The analysis of the variable influence on projection (VIP) of the OPLS-DA model was used to reveal features that were the most significant for the classification. A list of the lipids with VIP >1, a measure that can be used as cancer biomarkers, is summarized in Table S2.

**Figure 6:**
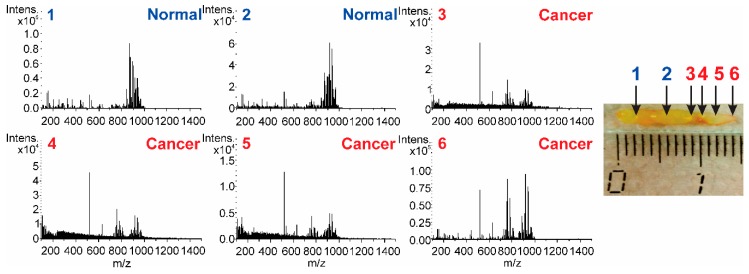
Positive-ion mass spectra at different points from one sample. Changes in MS profile correlate with shifting of the surgical probe from normal to tumor tissue. The figures in the photo indicate the points for which histological and LTEU-EESI-MS analyses were done.

Further validation of the OPLS-DA statistical model was done by classification of the LTEU-EESI-MS data from tissue samples containing both normal and cancer regions and their comparison with histological characterization. Several characteristic mass spectra from the sequential points are shown in Figure 6.

**Figure 7:**
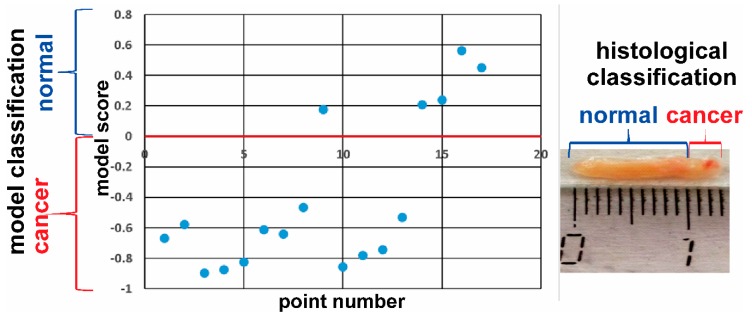
The plot of tissue classification score vs. its spatial position. The scores were obtained by unsupervised analysis of LTEU-EESI-MS mass spectra with the developed OPLS-DA model. The figures in the photo indicate several points for which histological and LTEU-EESI-MS analyses were done. The figures are colored according to histological results, with blue color corresponding to normal tissue and red color corresponding to cancer tissue. The red line on the graph was determined by statistical model and separates “normal region” from “cancer region”.

The estimated scores for several analyzed tissue points are plotted in Figure 7. Data points with negative ordinate correspond to normal-tissue profile. Data points with positive ordinate correspond to cancer-tissue MS profile. The bigger absolute values of the score are related to more pronounced tissue type. The switch between negative and positive scores corresponds to a margin between normal and cancer regions. Results obtained by OPLS-DA classification of the LTEU-EESI-MS data gave 97% sensitivity and 94% specificity as related to histological analysis. The mass spectra were obtained with 1 mm spatial resolution. Therefore, the estimated accuracy of tissue margin determination was not worse than 1 mm in the present study.

## 4. Conclusions

The LTEU-EESI-MS method provided fast and reliable differentiation of breast cancer and normal tissue with 97% sensitivity, 94% specificity, and spatial accuracy of at least 1 mm. Such high accuracy and spatial resolution of analysis were mainly achieved owing to the low temperature of the surgical tool and thus the small area of tissue thermal destruction. The combination of low-temperature electrosurgical unit and extractive electrospray ionization mass spectrometry is a promising method for intraoperative tissue characterization because it provides high molecular specificity and high stability and robustness of operation.

## Supplementary Materials

The following are available online, Figure S1: Optimization of the LTEU-EESI-MS ion source parameters, Table S1: Tentatively identified peaks based on accurate mass within 10 ppm, Table S2. Tentatively identified peaks with VIP ≥ 1. The identification is based on accurate mass within 10 ppm.

## References

[1] Mirnezami: R., Spagou K., Vorkas P.A., Lewis M.R., Kinross J., Want E., Shion H., Goldin R.D., Darzi A., Takats Z. (2014). Chemical mapping of the colorectal cancer microenvironment via MALDI imaging mass spectrometry (MALDI-MSI) reveals novel cancer-associated field effects. Mol. Oncol..

[2] Ye H., Mandal R., Catherman A., Thomas P.M., Kelleher N.L., Ikonomidou C., Li L. (2014). Top-down proteomics with mass spectrometry imaging: A pilot study towards discovery of biomarkers for neurodevelopmental disorders. PLoS ONE.

[3] Schäfer K.C., Szaniszló T., Günther S., Balog J., Dénes J., Keserü M., Dezsö B., Tóth M., Spengler B., Takáts Z. (2011). In situ, real-time identification of biological tissues by ultraviolet and infrared laser desorption ionization mass spectrometry. Anal. Chem..

[4] Woolman M., Ferry I., Kuzan-Fischer C.M., Wu M., Zou J., Kiyota T., Isik S., Dara D., Aman A., Das S. (2017). Rapid determination of medulloblastoma subgroup affiliation with mass spectrometry using a handheld picosecond infrared laser desorption probe. Chem. Sci..

[5] Woolman M., Gribble A., Bluemke E., Zou J., Ventura M., Bernards N., Wu M., Ginsberg H.J., Das S., Vitkin A. (2017). Optimized Mass Spectrometry Analysis Workflow with Polarimetric Guidance for ex vivo and in situ Sampling of Biological Tissues. Sci. Rep..

[6] Takats Z., Wiseman J.M., Gologan B. (2004). Mass Spectrometry Sampling Under Ambient Conditions with Desorption Electrospray Ionization. Science.

[7] Jarmusch A.K., Alfaro C.M., Pirro V., Hattab E.M., Cohen-Gadol A.A., Cooks R.G. (2016). Differential Lipid profiles of normal human brain matter and gliomas by positive and negative mode desorption electrospray ionization—Mass spectrometry imaging. PLoS ONE.

[8] Sans M., Gharpure K., Tibshirani R., Zhang J., Liang L., Liu J., Young J.H., Dood R.L., Sood A.K., Eberlin L.S. (2017). Metabolic markers and statistical prediction of serous ovarian cancer aggressiveness by ambient ionization mass spectrometry imaging. Cancer Res..

[9] Hu B., Lai Y.-H., So P.-K., Chen H., Yao Z.-P. (2012). Direct ionization of biological tissue for mass spectrometric analysis. Analyst.

[10] Kerian K.S., Jarmusch A.K., Pirro V., Koch M.O., Masterson T.A., Cheng L., Cooks R.G. (2015). Differentiation of prostate cancer from normal tissue in radical prostatectomy specimens by desorption electrospray ionization and touch spray ionization mass spectrometry. Analyst.

[11] Wei Y., Chen L., Zhou W., Chingin K., Ouyang Y., Zhu T., Wen H., Ding J., Xu J., Chen H. (2015). Tissue spray ionization mass spectrometry for rapid recognition of human lung squamous cell carcinoma. Sci. Rep..

[12] Pirro V., Seró R., Jarmusch A.K., Alfaro C.M., Cohen-Gadol A.A., Hattab E.M., Cooks R.G. (2017). Analysis of human gliomas by swab touch spray—Mass spectrometry: Applications to intraoperative assessment of surgical margins and presence of oncometabolites. Analyst.

[13] Kertesz V., Van Berkel G.J. (2010). Fully automated liquid extraction-based surface sampling and ionization using a chip-based robotic nanoelectrospray platform. J. Mass Spectrom..

[14] Zhang J., Rector J., Lin J.Q., Young J.H., Sans M., Katta N., Giese N., Yu W., Nagi C., Suliburk J. (2017). Nondestructive tissue analysis for ex vivo and in vivo cancer diagnosis using a handheld mass spectrometry system. Sci. Transl. Med..

[15] Sans M., Zhang J., Lin J.Q., Feider C.L., Giese N., Breen M.T., Sebastian K., Liu J., Sood A.K., Eberlin L.S. (2019). Performance of the MasSpec Pen for Rapid Diagnosis of Ovarian Cancer. Clin. Chem..

[16] Balog J., Szaniszló T., Schaefer K.-C., Denes J., Lopata A., Godorhazy L., Szalay D., Balogh L., Sasi-Szabó L., Toth M. (2010). Identification of biological tissues by rapid evaporative ionization mass spectrometry. Anal. Chem..

[17] St John E.R., Balog J., McKenzie J.S., Rossi M., Covington A., Muirhead L., Bodai Z., Rosini F., Speller A.V.M., Shousha S. (2017). Rapid evaporative ionisation mass spectrometry of electrosurgical vapours for the identification of breast pathology: Towards an intelligent knife for breast cancer surgery. Breast Cancer Res..

[18] Alexander J., Gildea L., Balog J., Speller A., McKenzie J., Muirhead L., Scott A., Kontovounisios C., Rasheed S., Teare J. (2017). A novel methodology for in vivo endoscopic phenotyping of colorectal cancer based on real-time analysis of the mucosal lipidome: A prospective observational study of the iKnife. Surg. Endosc. Other Interv. Tech..

[19] Taheri A., Mansoori P., Sandoval L.F., Feldman S.R., Pearce D., Williford P.M. (2014). Electrosurgery: Part I. Basics and principles. J. Am. Acad. Dermatol..

[20] Carr-Locke D.L., Day J. (2011). Principles of electrosurgery. Successful Training in Gastrointestinal Endoscopy.

[21] Helmut W., Jurgen L., Hans-Jurgen W., Rainer M., Hans-Dieter L. Characterization of tissue interaction by analyzation of electrosurgial smoke. Proceedings of the Annual International Conference of the IEEE Engineering in Medicine and Biology.

[22] Barrett W.L., Garber S.M. (2003). Surgical smoke—A review of the literature. Is this just a lot of hot air?. Surg. Endosc. Other Interv. Tech..

[23] Hensman C., Baty D., Willis R.G., Cuschieri A. (1998). Chemical composition of smoke produced by high-frequency electrosurgery in a closed gaseous environment An in vitro study. Surg. Endosc. Other Interv. Tech..

[24] Palanker D., Vankov A., Jayaraman P. (2008). On mechanisms of interaction in electrosurgery. New J. Phys..

[25] Balog J., Sasi-Szabo L., Kinross J., Lewis M.R., Muirhead L.J., Veselkov K., Mirnezami R., Dezso B., Damjanovich L., Darzi A. (2013). Intraoperative Tissue Identification Using Rapid Evaporative Ionization Mass Spectrometry. Sci. Transl. Med..

[26] Guenther S., Schäfer K.C., Balog J., Dénes J., Majoros T., Albrecht K., Tóth M., Spengler B., Takáts Z. (2011). Electrospray post-ionization mass spectrometry of electrosurgical aerosols. J. Am. Soc. Mass Spectrom..

[27] Schiavon M., Calabrese F., Nicotra S., Marulli G., Pozzato G., Giacometti C., Valente M., Rea F. (2007). Favorable tissue effects of quantum molecular resonance device (Vesalius) compared with standard electrocautery: A novel paradigm in lung surgery. Eur. Surg. Res..

[28] Demirhan E., Çukurova İ., Arslan İ.B., Ozkan E.T., Mengi E., Yigitbasi O.G. (2015). Quantum Molecular Resonance–Assisted Phonomicrosurgery: Preliminary Experience. Otolaryngol. Neck Surg..

[29] Schäfer K.C., Dénes J., Albrecht K., Szaniszló T., Balogh J., Skoumal R., Katona M., Tóth M., Balogh L., Takáts Z. (2009). In vivo, in situ tissue analysis using rapid evaporative ionization mass spectrometry. Angew. Chem. Int. Ed..

[30] Chen H., Venter A., Cooks R.G. (2006). Extractive electrospray ionization for direct analysis of undiluted urine, milk and other complex mixtures without sample preparation. Chem. Commun..

[31] Law W.S., Wang R., Hu B., Berchtold C., Meier L., Chen H., Zenobi R. (2010). On the Mechanism of Extractive Electrospray Ionization. Anal. Bioanal. Chem..

[32] Fahy E., Sud M., Cotter D., Subramaniam S. (2007). LIPID MAPS online tools for lipid research. Nucleic Acids Res..

[33] Liebisch G., Vizcaíno J.A., Köfeler H., Trötzmüller M., Griffiths W.J., Schmitz G., Spener F., Wakelam M.J.O. (2013). Shorthand notation for lipid structures derived from mass spectrometry. J. Lipid Res..

[34] Gu H., Xu N., Chen H. (2012). Direct analysis of biological samples using extractive electrospray ionization mass spectrometry (EESI-MS). Anal. Bioanal. Chem..

[35] Keller B.O., Sui J., Young A.B., Whittal R.M. (2008). Interferences and contaminants encountered in modern mass spectrometry. Anal. Chim. Acta.

