# Supplementary material for: Combination of Low-Temperature Electrosurgical Unit and Extractive Electrospray Ionization Mass Spectrometry for Molecular Profiling and Classification of Tissues
Source: Molecules. 2019 Aug 15;24(16):2957. doi: 10.3390/molecules24162957 (PMC6720730; doi:10.3390/molecules24162957)
Supplement: Supplementary file 1 [file molecules-24-02957-s001.pdf]

molecules

MDPI

Article

Combination of Low-Temperature Electrosurgical Unit and Extractive Electrospray Ionization Mass Spectrometry for Molecular Profiling and Classification of Tissues

Gennady Sukhikh [1,2], Vitaliy Chagovets [1], Xinchen Wang [3], Valeriy Rodionov [1], Vlada Kometova [1], Alisa Tokareva [4], Alexey Kononikhin [1,4], Natalia Starodubtseva [1,4], Konstantin Chingin [3], Huanwen Chen [3] and Vladimir Frankevich [1,*]

1 National Medical Research Center for Obstetrics, Gynecology and Perinatology named after Academician V.I.Kulakov of the Ministry of Healthcare of Russian Federation, Moscow 117997, Russian Federation
2 Department of Obstetrics, Gynecology, Perinatology and Reproductology, First Moscow State Medical University named after I.M. Sechenov, Moscow 119991, Russian Federation
3 Jiangxi Key Laboratory for Mass Spectrometry and Instrumentation, East China University of Technology, Nanchang 330013, China
4 Moscow Institute of Physics and Technology, Moscow 141701, Russian Federation
* Correspondence: vfrankevich@gmail.com

Table 1. Tentatively identified peaks based on accurate mass within 10 ppm.

#	Lipid	Exact mass	Accurate mass	Mass error, ppm	Adduct type
**1**	DG 24:6	467.2768	467.2768	6	Na^+
**2**	DG 24:7	465.2611	465.2611	4	Na^+
**3**	LPE 14:2	439.2568	439.2568	8	NH_4^+
**4**	LPE 18:6	487.2568	487.2568	5	NH_4^+
**5**	LPE O-22:6	534.2955	534.2955	2	Na^+
**6**	PC 32:0	734.5694	734.5694	5	H^+
**7**	PC 32:1	732.5538	732.5538	6	H^+
**8**	PC 34:0	784.5827	784.5827	6	Na^+
**9**	PC 34:1	760.5851	760.5851	4	H^+
**10**	PC 34:2	758.5694	758.5694	4	H^+
**11**	PC 36:0	812.614	812.614	3	Na^+
**12**	PC 36:1	788.6164	788.6164	3	H^+
**13**	PC 36:2	786.6007	786.6007	4	H^+
**14**	PC 36:4	782.5694	782.5694	4	H^+
**15**	PC 44:1	922.7235	922.7235	6	Na^+
**16**	PC 46:4	922.7259	922.7259	3	H^+
**17**	PE 34:1	718.5381	718.5381	7	H^+
**18**	PE 36:0	770.567	770.567	8	Na^+
**19**	PE 36:1	768.5514	768.5514	7	Na^+
**20**	PE 36:2	744.5538	744.5538	5	H^+
**21**	PE 38:0	798.5983	798.5983	5	Na^+
**22**	PE 38:1	796.5827	796.5827	5	Na^+
**23**	PE 38:2	772.5851	772.5851	4	H^+
**24**	SM 34:3	716.5701	716.5701	1	NH_4^+
**25**	SM 37:5	759.5411	759.5411	4	Na^+
**26**	TG 50:1	850.7858	850.7858	1	NH_4^+
**27**	TG 50:2	848.7702	848.7702	1	NH_4^+
**28**	TG 52:2	876.8015	876.8015	1	NH_4^+
**29**	TG 52:3	874.7858	874.7858	0	NH_4^+
**30**	TG 52:4	872.7702	872.7702	1	NH_4^+
**31**	TG 54:2	904.8328	904.8328	2	NH_4^+
**32**	TG 54:3	902.8171	902.8171	1	NH_4^+
**33**	TG 54:4	900.8015	900.8015	0	NH_4^+
**34**	TG 54:5	898.7858	898.7858	1	NH_4^+
**35**	TG 56:4	928.8328	928.8328	10	NH_4^+
**36**	TG 56:5	926.8171	926.8171	1	NH_4^+
**37**	TG 56:6	924.8015	924.8015	2	NH_4^+

**38**	TG 57:5	940.8328	940.8318	1	NH_4^+
**39**	TG 58:4	956.8641	956.8641	5	NH_4^+
**40**	TG 58:5	954.8484	954.8484	2	NH_4^+
**41**	TG 58:6	952.8328	952.8328	0	NH_4^+
**42**	TG 58:7	950.8171	950.8171	3	NH_4^+
**43**	TG 60:7	978.8484	978.8484	3	NH_4^+

Table 2. Tentatively identified peaks with VIP ≥ 1. The identification is based on accurate mass within 10 ppm. Lipids nomenclature is in accordance with LIPID MAPS [1] terminology and shorthand notation summarized in [2].

#	Lipid	Exact mass	Accurate mass	Mass error, ppm	Adduct type	VIP
**1**	PC 34:1	760.5851	760.5883	4	H^+	4.7
**2**	PC 34:2	758.5694	758.5725	4	H^+	3.3
**3**	PC 36:2	786.6007	786.6038	4	H^+	3.0
**4**	PC 36:3	784.5851	784.5873	3	H^+	2.8
**5**	PC 32:0	734.5694	734.5734	5	H^+	2.8
**6**	TG 56:4	928.8328	928.8235	10	NH_4^+	2.8
**7**	TG 54:3	902.8171	902.8164	1	NH_4^+	2.5
**8**	TG 56:5	926.8171	926.8177	1	NH_4^+	2.5
**9**	PC 36:1	788.6164	788.6142	3	H^+	2.5
**10**	DG 44:11	732.5562	732.5585	3	NH_4^+	2.5
**11**	PC 32:1	732.5538	732.5585	6	H^+	2.5
**12**	PC 36:4	782.5694	782.5723	4	H^+	2.3
**13**	TG 54:4	900.8015	900.8047	4	NH_4^+	2.3
**14**	TG 58:5	954.8484	954.8469	2	NH_4^+	2.3
**15**	TG 52:3	874.7858	874.7858	0	NH_4^+	2.2
**16**	TG 52:2	876.8015	876.801	1	NH_4^+	2.0
**17**	TG 58:6	952.8328	952.8325	0	NH_4^+	2.0
**18**	PE 38:2	772.5851	772.5884	4	H^+	2.0
**19**	PC 36:0	812.614	812.6168	3	Na^+	1.9
**20**	PE 36:2	744.5538	744.5577	5	H^+	1.9
**21**	TG 58:4	956.8641	956.8594	5	NH_4	1.9
**22**	SM 39:8	759.5436	759.5379	7	H^+	1.8
**23**	PE 38:4	790.5357	790.5295	8	Na^+	1.8
**24**	PE 34:1	718.5381	718.5432	7	H^+	1.8
**25**	PE 36:0	770.567	770.5729	8	Na^+	1.8
**26**	PE 38:1	796.5827	796.5864	5	Na^+	1.7
**27**	TG 52:4	872.7702	872.7707	1	NH_4	1.6
**28**	TG 56:6	924.8015	924.7999	2	NH_4	1.6
**29**	TG 54:2	904.8328	904.8306	2	NH_4	1.6
**30**	PE 38:0	798.5983	798.6025	5	Na^+	1.6
**31**	TG 54:5	898.7858	898.7866	1	NH_4	1.5
**32**	SM 44:0	862.7736	862.7823	10	NH_4	1.5
**33**	PE 36:1	768.5514	768.5568	7	Na^+	1.4
**34**	TG 50:2	848.7702	848.771	1	NH_4	1.3
**35**	SM 44:1	860.7579	860.7664	10	NH_4	1.2
**36**	TG 58:7	950.8171	950.8242	7	NH_4	1.0

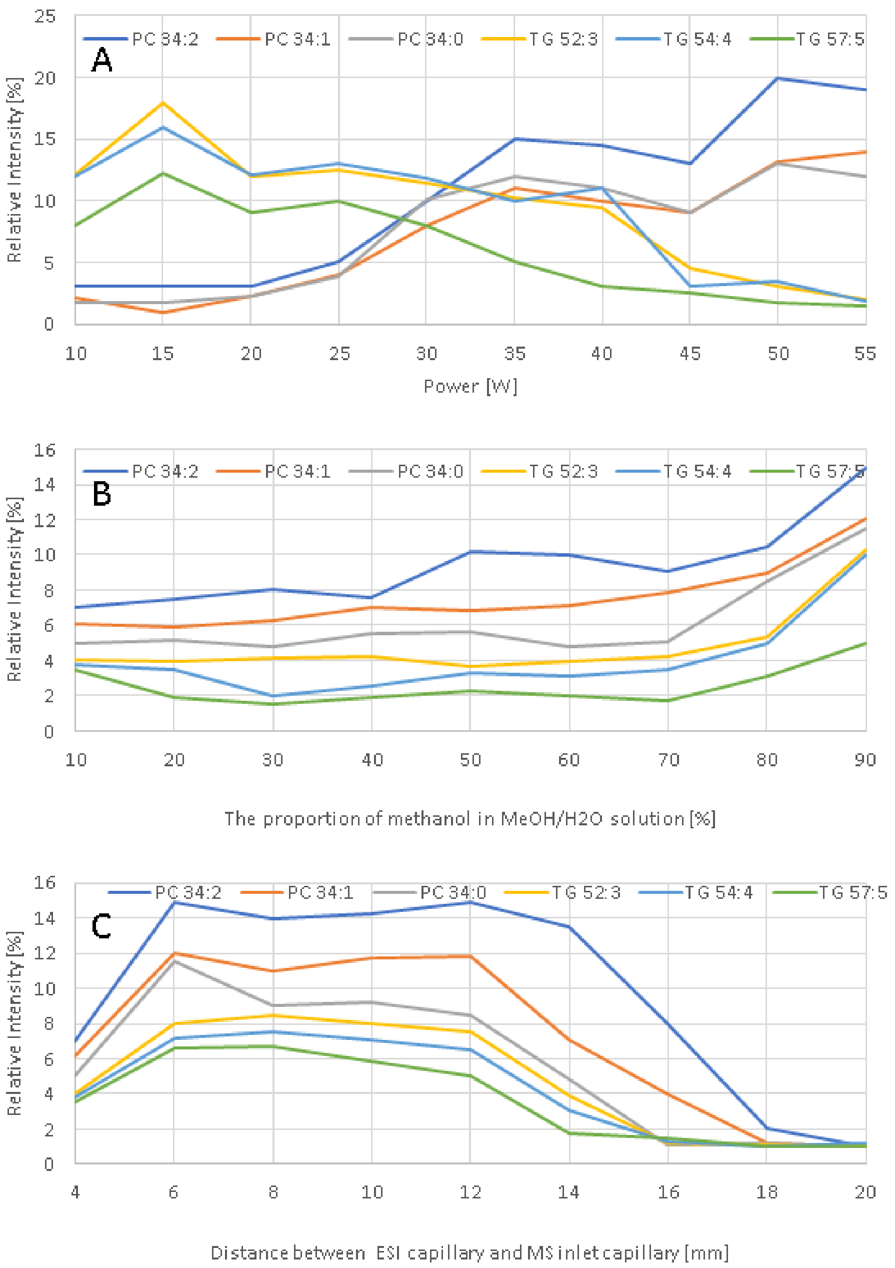

Figure 1. Optimization of the LTEU-EESI-MS ion source parameters. (A) Power of a surgical unit; (B) EESI solvent composition; (C) distance between ESI capillary and MS inlet capillary.
